# Stratigraphy of stable isotope ratios and leaf structure within an African rainforest canopy with implications for primate isotope ecology

**DOI:** 10.1038/s41598-021-93589-8

**Published:** 2021-07-09

**Authors:** B. E. Lowry, R. M. Wittig, J. Pittermann, V. M. Oelze

**Affiliations:** 1 Department of Anthropology, University of California Santa Cruz, 1156 High Street, Santa Cruz, CA 95060 USA; 2 Department of Ecology and Evolutionary Biology, University of California Santa Cruz, 1156 High Street, Santa Cruz, CA 95060 USA; 3 Max Planck Institute for Evolutionary Anthropology, Deutscher Platz 6, 04103 Leipzig, Germany; 4 Taï Chimpanzee Project, Centre Suisse de Recherches Scientifiques, B.P. 1303, Abidjan 01, Côte d’Ivoire

**Keywords:** Ecology, Biogeochemistry, Boreal ecology, Stable isotope analysis

## Abstract

The canopy effect describes vertical variation in the isotope ratios of carbon (δ^13^C), oxygen (δ^18^O) and partially nitrogen (δ^15^N) within plants throughout a closed canopy forest, and may facilitate the study of canopy feeding niches in arboreal primates. However, the nuanced relationship between leaf height, sunlight exposure and the resulting variation in isotope ratios and leaf mass per area (LMA) has not been documented for an African rainforest. Here, we present δ^13^C, δ^18^O and δ^15^N values of leaves (n = 321) systematically collected from 58 primate food plants throughout the canopy (0.3 to 42 m) in Côte d’Ivoire, West Africa. Besides leaf sample height and light availability, we measured leaf nitrogen and carbon content (%N, %C), as well as LMA (n = 214) to address the plants’ vertical resource allocations. We found significant variation in δ^13^C, δ^18^O and δ^15^N, as well as LMA in response to height in combination with light availability and tree species, with low canopy leaves depleted in ^13^C, ^18^O and ^15^N and slightly higher in %N compared to higher canopy strata. While this vertical isotopic variation was not well reflected in the δ^13^C and δ^15^N of arboreal primates from this forest, it did correspond well to primate δ^18^O values.

## Introduction

There is a remarkable amount of variation in the stable isotope ratios within even one organism. In plants growing in closed canopy forests, leaves measured at varying heights from one individual tree can have markedly different stable isotope ratios of carbon (δ^13^C), oxygen (δ^18^O) and to some extent even nitrogen (δ^15^N). This phenomenon has been widely described^1–8^ and is commonly referred to as the “canopy effect”^9^. Isotopic fractionation related to this effect is known to be the result of vertical gradients in sunlight, humidity, availability of atmospheric isotope values, source water and photosynthetic rates. Rather than height from the forest floor, the more ecologically meaningful way of thinking about the canopy effect is probably in terms of depth from the top canopy, as it is the dense structure of intertwining tree crowns that is largely causing these gradients rather than soil-mediated processes permeating upwards into the canopy^10^. However, by reason of feasibility, canopy effect studies have always considered height from the ground rather than estimating or measuring the depth from the top of the canopy. While most studies focused on δ^13^C variation within plants, some also included δ^18^O, δ^15^N and δ^2^H^7,9,11–17^. In this study we investigate plant δ^13^C, δ^18^O and δ^15^N in their relationship to the canopy effect and to stable isotope data of arboreal primate feeding at different canopy heights.

Several processes in the forest canopy cause fractionation and variation in δ^13^C. In the understory of closed canopy forests, air CO_2_ is reduced in ^13^C due to carbon recycling in the form of decomposing leaf litter on the forest floor, which affects the isotope composition of CO_2_ available for plants growing at different canopy heights^2,18,19^. Closed canopy forests will also experience significant gradients of photosynthetically available radiation (PAR) as the upper canopy can block between 95 and 99.5% of all light from ever reaching the forest floor^20^. In the lower canopy where lower light and temperature reduce transpiration rates, leaves can afford maximum stomatal opening. Here, diffusion of CO_2_ is high and the enzyme rubisco will preferentially fix ^12^CO_2_ while discriminating the heavier ^13^CO_2_. These leaves will have more negative δ^13^C values. However, in the upper canopy, where potentially high evaporative flux can lead to stomatal closure to conserve water, rubisco will fix ^13^CO_2_ at a higher rate than in the understory. Hence, upper canopy leaves will have higher δ^13^C values^21^ (and references therein). The canopy effect is often measured in forests with very large trees, such as in large conifers, which will compensate for hydraulic constraints due to gravity by modifying various traits associated with water transport and photosynthesis^22–24^.

Variation in δ^18^O within the forest canopy is mainly driven by atmospheric humidity levels. Low relative humidity in the upper canopy tends to lead to higher rates of transpiration^7,14^. Preferential loss of lighter H_2_^16^O during evaporation will leave leaf tissue enriched in remaining ^18^O. Besides rates of evaporation, the δ^18^O composition of plant water is primarily determined by the isotope composition of source water in the soil, which vary geographically and is affected by local surface air temperature, altitude, amount of precipitation, etc.^12,25,26^. The canopy effect in δ^18^O, with δ^18^O values varying with canopy height, has been reported for tropical forest ecosystems with closed canopy cover^7,14^, yet not all studies could find a clear difference between understory and canopy leaves δ^18^O^11^.

δ^15^N values in non-leguminous plants are determined by the isotopic composition of the source soil^27^. δ^15^N is predicted to fluctuate between ecological zones based on variation in temperature and precipitation as well as mycorrhizal activity^27^. While epiphytes are seen to experience different rates of δ^15^N fractionation with canopy height^16^, fractionation of δ^15^N within individual leaves has not been seen to be affected by height in trees^11^. In this study, we explore whether leaf δ^15^N values vary with canopy height, which has only been documented in a few cases^8^.

The considerable gradient in light intensity within the canopy does not only affect isotope fractionation, but also the chemical and structural makeup of the leaves, such as leaf mass per area (LMA). Similar to the canopy effect in isotopes, leaf structure varies with canopy height to maximize radiation-use efficiency and photosynthetic rate with varying levels of light intensity within the canopy. Understory leaves experiencing shade conditions will maximize their radiation-use efficiency by compensating with larger, thinner leaves (lower LMA) to increase photon capture^7,21,28^. In shade leaves, nitrogen is preferentially allocated to components of light-harvesting processes, whereas sun leaves invest nitrogen into rubisco. However, with increasing height, hydraulic limitations due to gravity, and water stress imposed by greater vapor pressure deficit, leaves will increasingly invest in water saving strategies to optimize water use efficiency^7,29^. These high canopy leaves tend to be smaller than their shade-adapted counterparts, with more numerous and larger cells to increase leaf capacitance, altogether making the leaf thicker (higher LMA) and thus more resilient to water loss^21^. This variation in LMA within the forest canopy should have consequences for the material properties and nutritional value of leaves as well as herbivore food preference^30,31^.

The predictability of the canopy effect in the stable isotope ratios of forest plants allows for the study of feeding and foraging behavior in elusive or extinct species of primates and arboreal animals by means of stable isotopes^32,33^. Isotopic variation in leaves and fruits due to height differences in the canopy can be expected to be reflected in the hair, bones, and teeth of primates which feed on these various plant food items^34,35^. In tropical forests around the world, arboreal primate species occupy specific spatial niches within the vertical strata of the canopy^36^^(and references therein)^. Long term field studies have observed primate species of the genera *Colobus, Cercopithecus, and Cercocebus* displaying habitat preference within the canopy of the rainforest of Taï National Park in Côte d’Ivoire, with different sympatric primates occupying different strata within the rainforest canopy. Body size constraints and dietary niches seem to drive spatial differentiation for Taï’s arboreal primates, and this is most pronounced during inter-specific associations^37^. Variations in branch size and stability as well as plant material properties and possibly uneven nutrient availability within the canopy are assumed to correlate with a vertical stratification of feeding niches occupied by different sympatric arboreal primate species^34^. An isotope study carried out in Taï National Park, measured the δ^13^C and δ^18^O values in primate bone collagen and apatite to determine the extent to which isotopes reflected this stratification in the canopy^34^. While variation in δ^13^C values was in large part a reflection of mixed diets rather than height in the canopy^33,38^, the study found strong correlation between δ^18^O values and canopy strata use. This is interesting as while δ^13^C and δ^15^N mainly relate to the isotopic characteristics of ingested foods, δ^18^O in animal bodies can be determined by not only the food, but much more so by drinking water and air^39^. Arboreal primates are commonly assumed to mainly rely on water deriving directly from plant foods^40^. This would suggest that leaf δ^18^O values will considerably affect the δ^18^O values of these primates, which we will test in this study.

In this study, we explore the canopy effect in plant foods which is underlying the isotopic variation found in these arboreal primates, by reporting bulk organic δ^13^C, δ^18^O and δ^15^N baseline values in 321 leaves of 58 individual plants collected at a range of heights between the understory and emergent trees (0.3 to 42 m) of Taï National Park (Table 1). These consist of 12 plant species for which long-term observation records show that they are particularly relevant for the diet of both chimpanzees (*Pan troglodytes versus*) and the different arboreal primates of Taï forest^37,41^ (unpublished data, Boesch & Wittig, personal communications McGraw). We here aim at establishing a multi-isotope baseline for future research on canopy stratigraphy and arboreal feeding niches in this and other rainforest ecosystem, which has the potential to also inform paleoecological reconstructions of forest species communities in the fossil record^33,42^.

**Table 1:** Overview of leaf samples analyzed in this study.

Plant species	Plant type	n individuals	Total n leaf samples
*Afzelia bella*	Emergent tree	5	29
*Dialium aubrevilli*	Emergent tree	6	47
*Parkia bicolor*	Emergent tree	5	35
*Sterculia oblonga*	Emergent tree	4	27
*Coula edulis*	Midcanopy tree	5	44
*Diospyrus canaliculata*	Midcanopy tree	5	28
*Musanga cecropioides*	Midcanopy tree	6	24
*Landolphia foretiana*	Vine	5	25
*Piper guinensis*	Vine	5	28
*Glyphea brevis*	Understory	5	16
*Urera oblongifolia*	Understory	2	7
*Sarcophynium brachystachys*	Understory	5	11
Total		58	321

We predicted that the leaves of the tallest trees, exposed to higher proportions of radiation, will modify their structure to accommodate for high rates of photosynthesis and evaporation. As a result of this strategy and other factors summarized under the canopy effect (e.g. increase in humidity, changes in availability of atmospheric vs. respirated CO_2_), leaf δ^13^C and δ^18^O values will strongly correlate with sampling height in interaction with light (PAR), producing the lowest isotope values in the forest understory and the highest values in leaves of emergent trees^5,6^. We had no such clear predictions for leaf δ^15^N values, as few studies found any significant variation in δ^15^N with height in the canopy^8,16^. Further, we investigated how leaf height and light availability affect leaf structure in an effort to better understand and describe the underlying mechanisms associated with isotopic fractionation. We predicted that LMA measurements (n = 214) will be lowest in understory plants and highest in emergent trees. We further anticipated that nitrogen content (%N) of leaves may be negatively correlate with LMA as smaller more shaded leaves should invest into higher photosynthetic capacity. Finally, we considered how leaf mass and %N may have implications for the material properties of leaves and thus relevance for primate feeding behavior.

## Results

**Figure 1:**
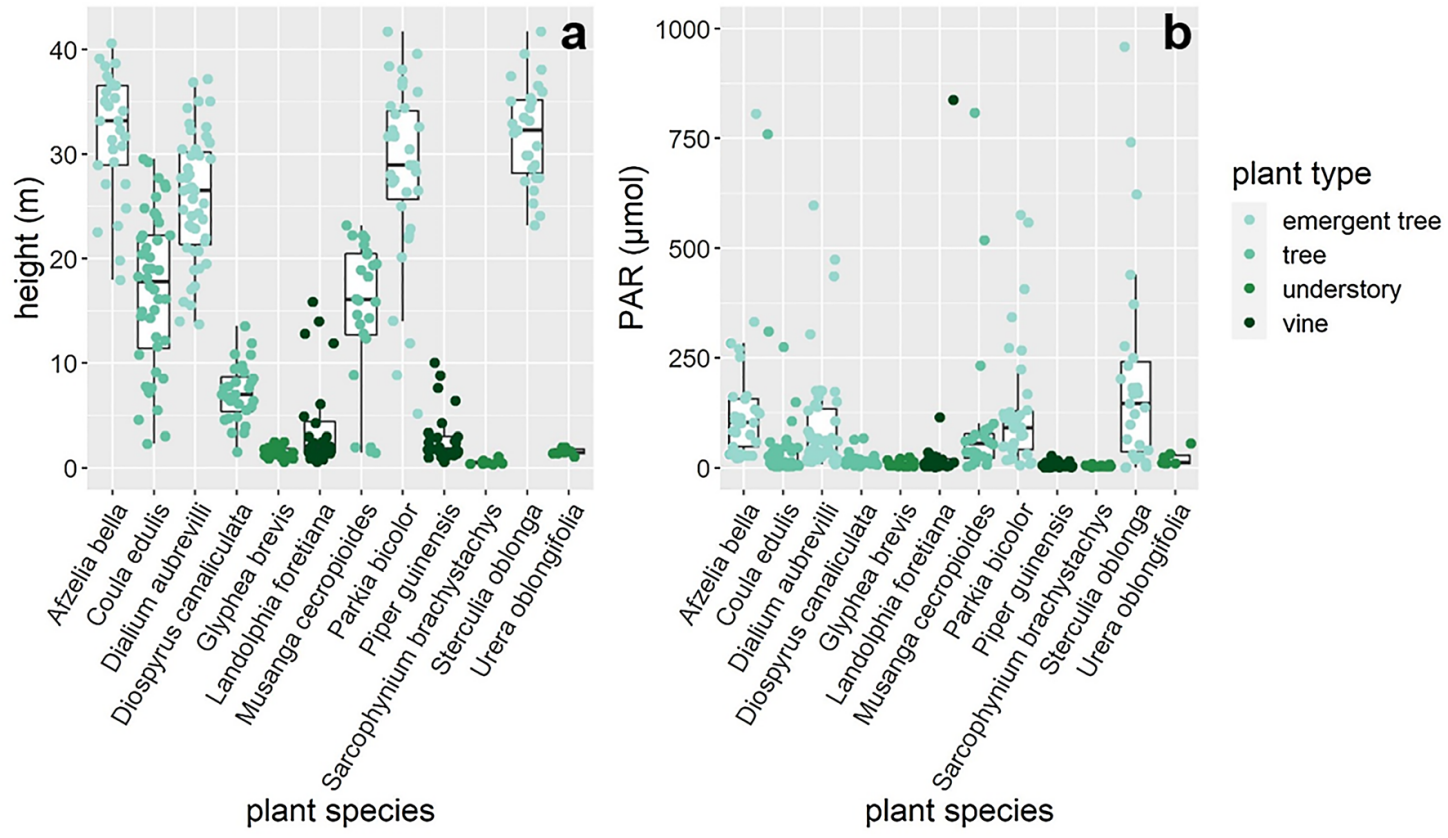
Whisker plots illustrating the effect of plant species on (**a**) leaf sample height and (**b**) photosynthetic active radiation (PAR).

**Figure 2:**
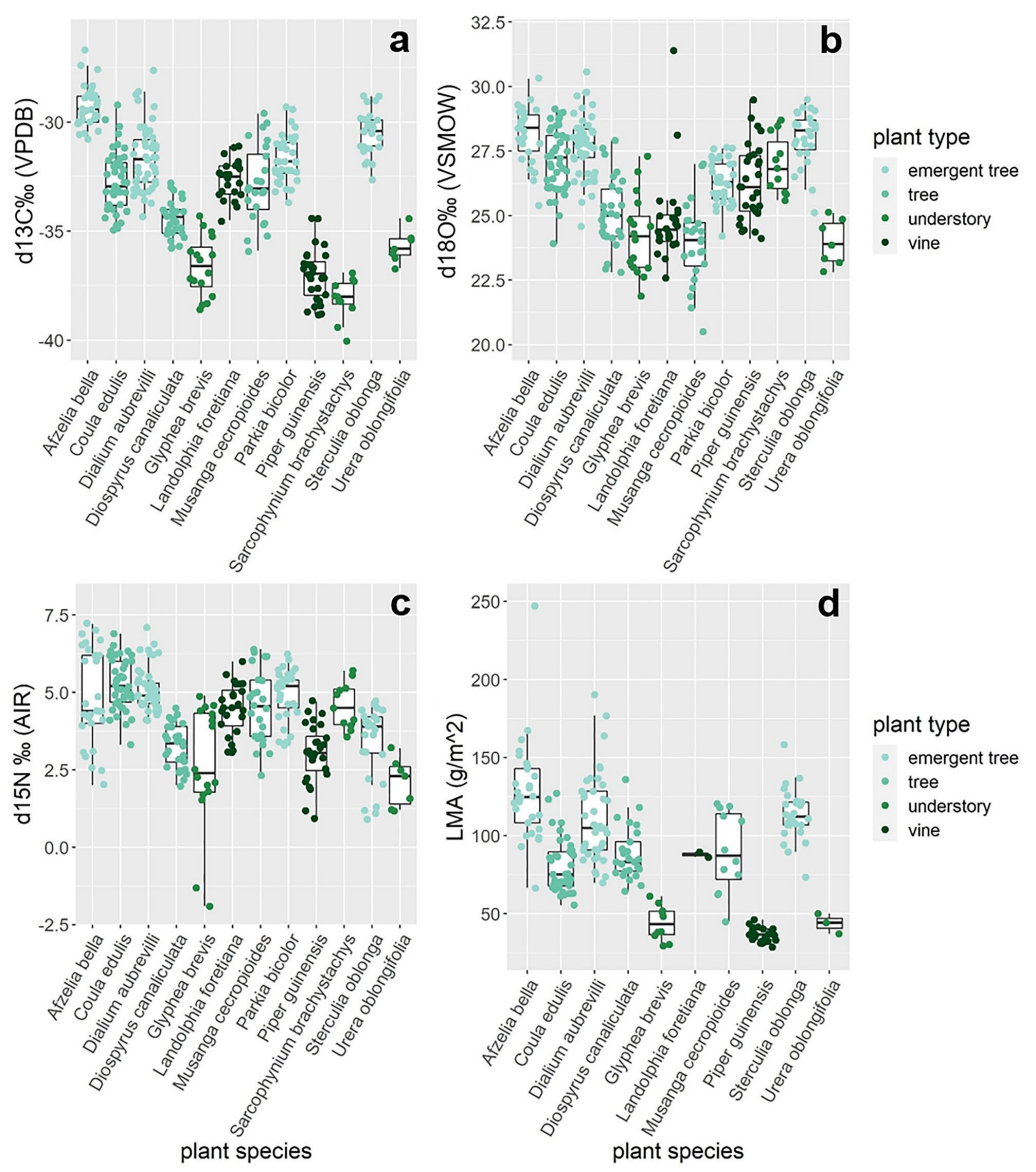
Whisker plots illustrating the effects of tree species on the variation in (**a**) δ^13^C, (**b**) δ^18^O, (**c**) δ^15^N and (**d**) PAR values within leaves.

We collected 321 leaves from 58 understory plants, vines, mid canopy trees and emergent trees of 12 species at ~ 1 m intervals ranging from the forest floor up to the highest point in the canopy (42 m) and documented considerable variation in PAR (Fig. 1). In this sample, we recorded considerable variation of δ^13^C (− 40 to − 26.7‰, mean = − 32.9‰), δ^18^O (20.5 to 31.4‰, mean = 26.4‰) and δ^15^N values (− 1.9 to 7.2‰, mean = 4.3‰). LMA values (mean = 90.8 g/m^2^) varied considerably between samples, ranging from 28.6 to 247.1 g/m^2^ (Fig. 2a–d, Table S1).

**Figure 3:**
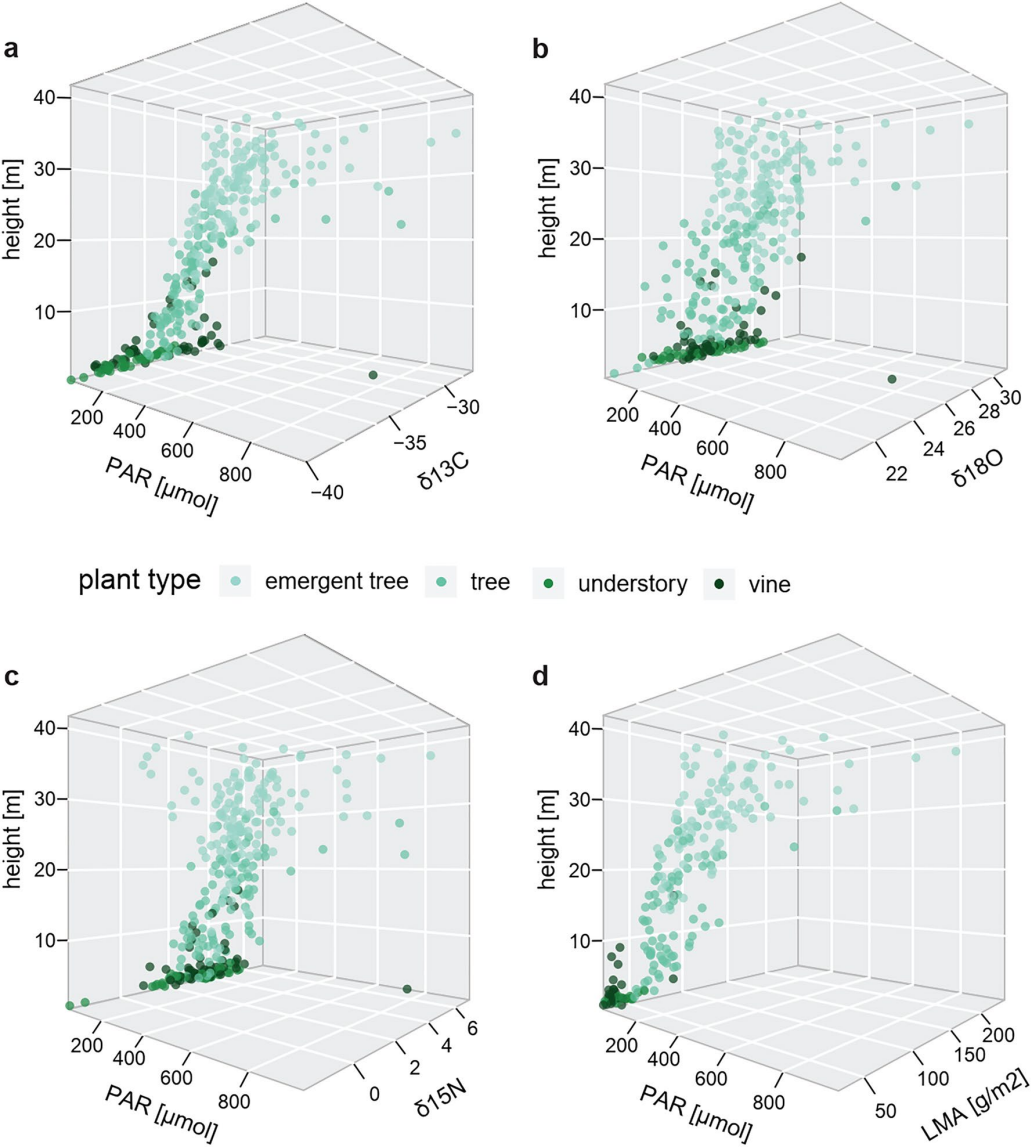
3-D plots illustrating the joint effects of sampling height (m) and PAR (umol) within the forest canopy on the (**a**) δ^13^C (VPDB), (**b**) δ^18^O (VSMOW), (**c**) δ^15^N (AIR), as well as (**d**) LMA values of leaves in μmol m^−2^ s^−1^. Different data point colors relate to different plant types within the plants sampled in this study. The single outlier data point from the vine species *Landolphia foretiana* (Lan.2.1) was excluded from our statistical data analysis.

We ran four models to test the effects of height (m) in interaction with PAR (μmol m^−2^ s^−1^), tree species, %N and %C and the random effect of plant individual on the response variables δ^13^C, δ^18^O, δ^15^N (‰) and LMA (g/m^2^). All models were highly significant, suggesting that height and PAR, or the interaction of both, as well as %N, %C and tree species have significant effects on δ^13^C, δ^18^O and δ^15^N values and LMA (Table 2, see Table S2 for detailed model results). The δ^13^C model was highly significant (χ^2^ = 417.3, df = 16, p < 0.0001) and showed that while %C was not significantly affecting δ^13^C values, the effect of height interacting with PAR was significant and tree species was highly significant, with higher and more light exposed leaves revealing significantly higher δ^13^C values (see Fig. 3a). The δ^18^O model was also highly significant (χ^2^ = 108, df = 16, p < 0.0001). Here the main driving effects were again sample height interacting with PAR and also tree species, with increasing height and PAR resulting in significantly higher δ^18^O values (Fig. 3b). The δ^15^N model without an interaction between sample height and PAR was significant as well (χ^2^ = 76, df = 15, p < 0.0001). Here, tree species was highly significant, whereas %N and the effect of PAR were significant, with lower light exposure resulting in lower leaf δ^15^N values (Fig. 3c). Sampling height had no effect on δ^15^N values of leaves and neither did %C. Finally, the LMA model was also highly significant (χ^2^ = 214, df = 14, p < 0.0001). Here the main driving effects were identified to be particularly %N, tree species and the interacting covariates height and PAR. Increasing height and PAR consistently lead to higher LMA and reductions in %N across plant species (Fig. 3d). The variable %C however had no effect on the LMA of leaves in this study (Table 2).

**Table 2:** Model results for each of the four linear mixed models.

Model	Predictors	npar	AIC	LRT	p-value
δ^13^C		#	793	#	#
	%C	1	792	1.34	0.247
	%N	1	797	6.16	**0.013**
	Plant species	11	847	76.70	**0.000**
	Height : PAR	1	801	10.63	**0.001**
δ^18^O		#	1041	#	#
	%C	1	1040	1.00	0.318
	%N	1	1041	2.38	0.123
	Plant species	11	1097	77.94	**0.000**
	Height : PAR	1	1045	5.91	**0.015**
δ^15^N		#	733	#	#
	Height	1	735	3.70	0.054
	Light	1	737	5.92	**0.015**
	%C	1	733	1.80	0.180
	%N	1	746	14.72	**0.000**
	Plant species	11	757	45.52	**0.000**
LMA		#	− 141	#	#
	%C	1	− 142	0.38	0.540
	%N	1	− 117	25.67	**0.000**
	Plant species	9	− 130	29.18	**0.001**
	Height : PAR	1	− 136	6.72	**0.010**

Significant effects are highlighted with p-values in bold.

## Discussion

For plants growing in a competitive regime, strategies which maximize light efficiency or water efficiency will result in very different structural and nutrient partitioning strategies which will likely have consequences for isotopic fractionation within plant parts. This study documents the gradual changes in leaf isotope ratios in response to absolute height and the sunlight available for photosynthesis and responsible for leaf water evaporation, and thus provides further evidence on the so-called canopy effect in δ^13^C, δ^18^O and even δ^15^N.

First, height and evapotranspirative demand are known to impact carbon and oxygen discrimination in leaves along the canopy height gradient. Leaf water potentials decrease with increasing elevation due to longer water transport path lengths, as well as the downward pull of gravity on the water column^29,43^. Consequently, upper canopy leaves will reduce their stomatal conductance in response to the perceived lower water availability^23^. Furthermore, leaves will also reduce stomatal conductance under increased vapor pressure deficit, brought about by the lower humidity levels typically experienced in the higher strata of the forest canopy^21,44^.

Also, light availability shapes the structural and functional trade-offs of leaves growing at the top of the canopy as well as in the understory. The level of radiation able to permeate the dense upper canopy will play an important role in determining understory productivity so it is important to account for heterogeneity in light composition in the understory by measuring PAR at consistent intervals in the canopy^45^. Solar radiation is a driver of photosynthetic rates, relative humidity levels and evaporation rates in the forest interior. Indeed, δ^13^C and δ^18^O values as well as measures of LMA, all of which are highly influenced by photosynthetic rate and evaporation respectively, were highly correlated with PAR (Fig. 3a,b,d). Emergent trees in our dataset consistently received the highest levels of PAR; leaves sampled from these trees also had the highest δ^13^C values, as well as some of the highest values in δ^18^O, δ^15^N and LMA. Similarly, samples obtained from understory plants received some of the lowest levels of PAR and subsequently show the lowest values of δ^13^C, δ^18^O, δ^15^N and LMA (Figs. 2 and 3). These results are consistent with our prediction that the tallest trees, which are able to capture high proportions of radiation incident on a tree canopy, will modify their leaf structure to account for high rates of photosynthesis and evaporation and will be enriched in ^13^C and ^18^O respectively^13,21^.

Our measurements of δ^13^C values in leaf tissue were highly correlated with the dynamic effect of height interacting with light (Fig. 3a). We found a large variation of δ^13^C values within the canopy of this forest [− 40 to − 26.7‰] also in accordance to the higher rates of CO_2_ cycling occurring in leaves lower in the canopy due to lower evaporative stress and higher stomatal conductance^46^. In general, the δ^13^C values we report here are more negative than the δ^13^C variation previously found in rainforest canopies^2,7,11,47,48^ with the exception of a few understory plants in the Congo Basin^49^. These particularly low δ^13^C values in the understory of Taï forest could be explained by unusually low rates of air circulation and high rates of recycling of respiratory CO_2_^1,50^. Given that high humidity levels should also increase isotopic fractionation in the forest understory^13^. It should be noted that field sampling took place during the relatively wet rainy season and daily humidity levels were high.

Measurements of bulk leaf material δ^18^O values were also highly predicted by the interacting effects of height and light in the canopy (Fig. 3b). While some previous work found similar canopy effects in closed canopy forest plant δ^18^O^7^, others were not able to reproduce this pattern in smaller and more diverse plant part samples^11^. The variation in our measurements of δ^18^O values of these leaves throughout the canopy was considerable [20.5 to 31.4‰] and δ^18^O values were more negative as compared to past studies (Table S1)^7,11^, possibly because Taï forest may experience higher average temperatures and evapotranspiration compared to other field sites.

**Figure 4:**
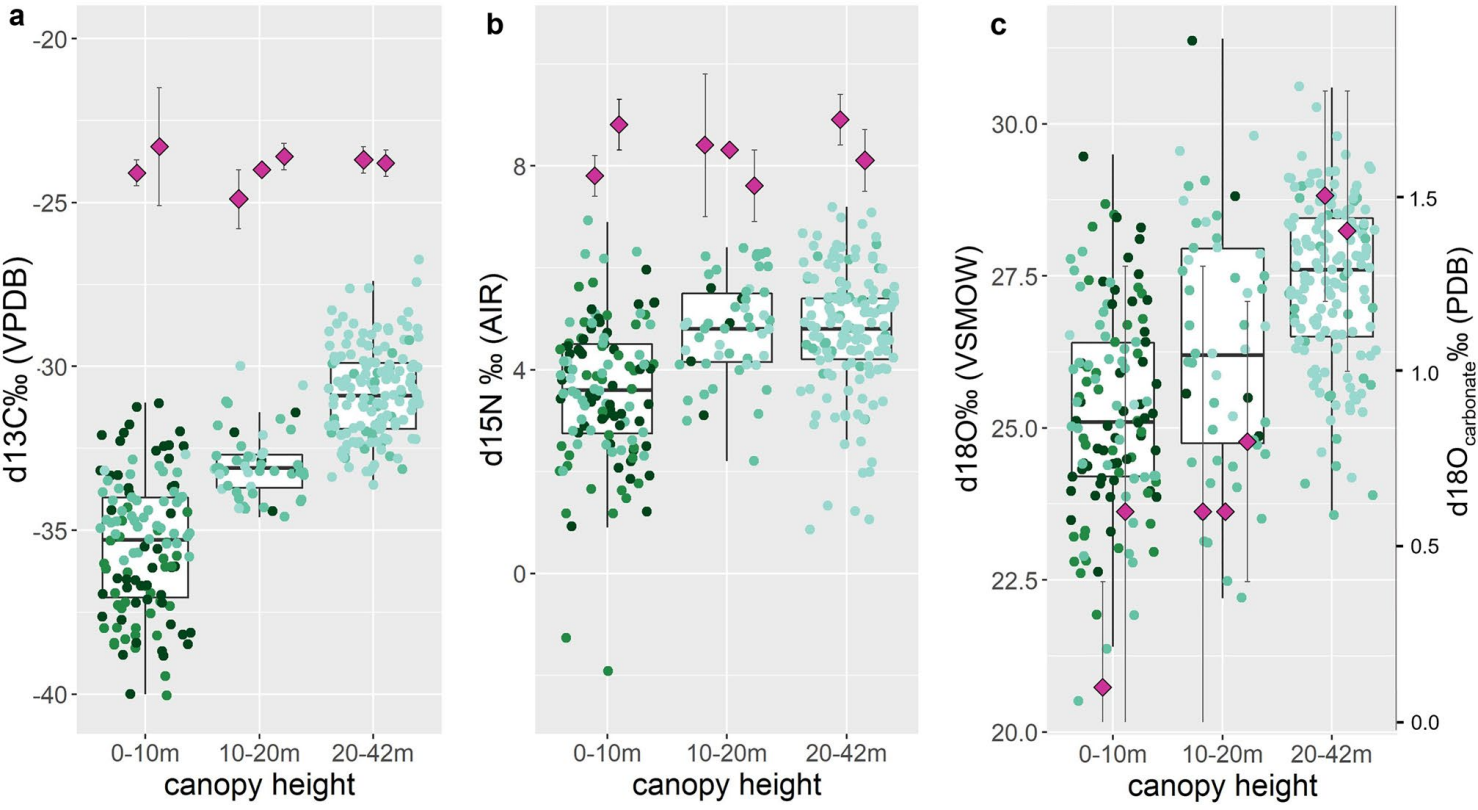
Whisker plots showing the distribution of (**a**) δ^13^C, (**c**) δ^15^N, and (**b**) δ^18^O values in leaves of different plant types (legends Figs. 1, 2, 3) across the canopy strata occupied by arboreal primate species at Taï forest. Average primate isotope data from bone collagen (**a**,**b**) and carbonate (**c**) are shown by canopy strata use as pink diamonds (± 1σ SD). The low canopy is most used by the taxa *Cercocebus atys* and *Cercopithecus campelli*, mid canopy is most relevant for *C. petaurista*, *C. diana* and *Procolobus verus*, whereas high canopy levels are inhabited by *Procolobus badius* and *Colobus polykomos*^37^. Vertical niche differentiation is best reflected by δ^18^O.

While previous research found no or only an inconsistent positive effect of height in δ ^15^N values^8,11,16,17^, we found a significant correlation between PAR and δ^15^N values, as well as a larger within-canopy variation in δ ^15^N values than reported so far. However, we did not find a significant relationship between δ ^15^N values and height directly, suggesting that variation in δ^15^N values may not be linked to what we call the canopy-effect per se (Figs. 2c, 4b). Our understanding so far is that δ ^15^N values in plants are highly dependent on the δ^15^N ratios of the source soil as well as any symbiotic associations rather than height or hydraulic limitations^51^. Ometto and colleagues^8^ found slight, but significantly lower understory δ^15^N values compared to the higher forest layers in two out of four sites measured in the Amazon, suggesting this may be related to different nitrogen sources in the soil, e.g. through the higher uptake of NO_3_ by understory vegetation. Height also affected δ^15^N values in epiphytes but was assumed to be associated to fractionation during nitrogen acquisition and mycorrhizal associations and possibly also related to water stress^16^. As in our study height was not a driving factor in leaf δ^15^N variation, we assume that PAR related evaporation stress may partly explain the fractionation of δ^15^N in leaves, and less so nitrogen source.

Low levels of light in the understory have profound impacts on light acquisition morphology of “shade leaves”, which tend to have higher levels of chlorophyll, and larger, thinner leaves to improve light capturing efficiency^21,52^. We found a significant correlation between leaf LMA and height (Fig. 3d), as well as significant variation in LMA between plant species (Fig. 2d). Leaves of vines and understory plants had consistently lower LMA measures compared to emergent and midcanopy trees. Emergent and midcanopy trees will experience higher degrees of water stress due to gravity and evaporation, especially in the upper canopy, and will adjust their leaf physiology to conserve water loss. However, vines and understory plants experience less water stress and may be less likely to grow leaves suited for water conservation^21^. The relationship between leaf toughness and height has implications for primate food preference as primates can be assumed to select for high energy yields which involve the least handling and masticatory investment^53^. Thus, primates may prefer leaves with low LMA in the lower canopy, which are thinner, presumably less tough and hence require less mastication energy over high canopy leaves^30^. High canopy leaves or “sun leaves” are expected to have higher toughness and require higher energy usage to consume^30,54^. In their 2008 study, Onoda and colleagues measured leaf toughness as a product of LMA and “punch strength” or maximum force/area and found that the toughness of sun leaves was mainly determined by their high LMA. The correlation we found between height, PAR and LMA in leaves from Taï forest makes LMA an interesting parameter for future work on the canopy effect and primate isotope ecology.

We also found a highly significant negative correlation between LMA and %N (Table 2) in our leaf samples. Low light leaves invest more nitrogen into light harvesting pigments, such as chlorophyll, for greater light capturing efficiency^28^. Alternatively, leaves in the upper canopy experience light-saturated rates of photosynthesis and will invest in enzymes associated with carbon fixation, mainly rubisco, to enhance their photosynthetic capacity^21^. Hence, our findings also suggest that leaf %N varies indirectly with height, which may affect primate feeding behavior. Primates are thought to prefer leaves with high protein content^55^. Since fruits are low in protein, primates not obtaining protein from invertebrate or vertebrate foods, will rely on leaves for a large portion of the protein in their diet and may preferentially select foliage high in nitrogen^56,57^. While this variation in %N is minimal, ranging from ~ 1 to 3.5 weight%, it might be of relevance to folivorous primates in combination with LMA and leaf toughness (see below).

Primates living in Taï National Park, grossly group into the feeding niches low (> 10 m, *Cercocebus atys* and *Cercopithecus campelli*), mid (10–20 m, *C. petaurista*, *C. diana* and *Procolobus verus*) and high (< 20, *Procolobus badius* and *Colobus polykomos*) canopy, which differ clearly in the stable isotope ratios of leaves (Fig. 4). Our leaf isotope data would suggest that feeding heights should be most clearly detectable in primate δ^13^C and δ^18^O values and could even have a slight effect on δ^15^N. However, Fig. 4 shows that the isotope signatures of Taï primates feeding at different height categories do not match well with those in leaves in δ^13^C or δ^15^N, but they do correspond well to the leaf δ^18^O values. The δ^13^C canopy effect is barely reflected in the collagen and apatite δ^13^C values of seven different arboreal primate species within Taï forest^34^. This notion, that feeding behaviors will substantially shift the δ^13^C values from what we would expect from mammals living in a closed canopy rainforest, is now well described for African and Amazonian rainforest^58^. Primates feeding in the understory and shrub layer up to 10 m heights have δ^13^C values similar to primates feeding in the highest canopy although understory leaves are on average ~ 5‰ lower in δ^13^C. In fact, all primates measured by Krigbaum and colleagues^34^ have bone collagen δ^13^C values much higher than the foliage in their canopy strata would predict (Fig. 4a). This can be explained by the systematic differences in δ^13^C between photosynthetic (leaves) and heterotrophic (flowers, fruits, seeds, bark, stem) plant parts, with leaves always being several ‰ lower in δ^13^C compared to other plant organs^17,59^. We can expect that fruit and seed eating primates will always have higher δ^13^C values than their folivorous counterparts, even if they forage in the forest understory such as sooty mangabeys (*Cercocebus atys*). And indeed, the most folivorous primate at Taï, the olive colobus monkey (*Procolobus verus*) had the lowest δ^13^C values among all taxa^34^. In sum, the degree of folivory versus frugivory should be accounted for when using δ^13^C in reconstructing canopy strata use^60^.

Our study suggests that δ^18^O has the potential to be a much more reliable tool in reconstructing canopy feeding niche than δ^13^C because δ^18^O values in plant foods will vary significantly with height^7,14^ (Fig. 4b) without the systematic isotopic offsets between leaves and heterotrophic plant parts we see in δ^13^C^11^. This suggests that the δ^18^O data should correspond well to the δ^18^O values of primate consumers. Indeed, the best predictor for feeding height in primate skeletal remains was previously shown to be δ^18^O, irrespective of the degree of folivory or frugivory^34,60^. We can confirm this here when we compare our results with the carbonate δ^18^O values of arboreal primates feeding at different heights in the same forest (Fig. 4c). This makes this isotope system particularly interesting for canopy feeding niche reconstructions in primates^38,61^. It is possible that atmospheric O_2_ and water vapor, other important sources of body water^39^, may also vary throughout the different strata of the forest canopy and contribute to this observed pattern. This will remain to be tested. Rainwater accumulations in tree holes may be an additional source of water undergoing different rates of evaporation in the forest canopy, though their use has not been described for the primates mentioned in this study^40^.

Our data suggest that δ^15^N has the potential to gain relevance in the study of canopy niches as well. When we group the δ^15^N values into three categories low (0–10 m), mid (10–20 m), and high (20–42 m) as shown in Fig. 4b, it becomes clear that mid and high canopy leaves are indistinguishable in δ^15^N, but low canopy leaves are on average ~ 1‰ lower in δ^15^N (mean 3.6 ± 1.4‰ 1σ) than leaves higher than 10 m (4.7 ± 1.2 δ^15^N‰ 1σ). However, this effect is barely visible, e.g. in the δ^15^N values of low canopy foraging primates at Taï (see Fig. 4b), such as the sooty mangabeys and diana monkeys (*Cercopithecus diana*)^34^. Nevertheless, that canopy height may affect δ^15^N values of plant foods may find some consideration in future isotopic research as an effect which may interfere with interpretations of faunivory and trophic level^62^.

This isotope study on the canopy effect in primate foods is limited to mature leaves out of logistical reasons as the timing of flowering, fruiting and young leaf flushing can be unpredictable and stretch over many months. Canopy research however, is extremely risky and should involve the presence of experienced certified arborists, which rarely are available for fieldwork months on end. Nevertheless, we are confident that similar isotopic effects we describe for leaves will also likely be found in other forest plant foods. Non-photosynthetically active plant parts such as fruits, flowers and seeds can be expected to source their water and energy from proximate sources within the plant, e.g. from adjacent leaves via phloem translocation^63^. A previous study on the canopy effect suggested that young and mature leaves do not substantially differ in δ^13^C or δ^18^O and mature foliage and fruits did not differ in δ^18^O^11^. On the other hand, isotopic fractionation between specific plant organs are well described for δ^13^C and allows us to correct plant δ^13^C data for plant part^17,59^. In sum, it is important to understand that both plant part and canopy height can be expected to significantly affect the δ^13^C ratios of plant foods, whereas canopy height and water source will be the stronger predictor for δ^18^O. This suggests that sampling of various primate plant food species remains crucial for research in primate isotope ecology when focusing on nuanced effects of canopy niche and dietary adaptations.

## Methods

This study was conducted in compliance with all relevant institutional, national, and international guidelines and legislation. None of the plant species selected for this study are listed by the IUCN as endangered species. Voucher specimens (for IDs see Table S1) identified by F. G. Gnimion, are stored and publically available at the Primate Ecology and Molecular Anthropology Laboratory at the University of California Santa Cruz. Field work was conducted under research authorization permit 067/MESRS/DGRI/DR issued by the *Ministre de l'Enseignement supérieur et de la Recherche scientifiqu*e, Côte d’Ivoire. We collected mature leaves at ~ 1 m increments for 58 individuals of 12 different plant species (total n = 321) consumed by primates in the research area of the Taï Chimpanzee Project, located in the primary rainforest of Taï National Park, Côte d’Ivoire, West Africa (Table 1, also see Supplementary Materials). Sampling was mostly accomplished by using rope access tree climbing techniques^64^ under the support of arborist James Luce. For each leaf sample we identified and recorded species, plant ID, date, time of day and %cloud cover. During sampling we measured exact height (m) with a measuring tape lowered to the forest floor and PAR with a handheld quantum light sensor (see Table S1 for the full dataset and SI for detailed sampling description). All samples were imported to the USA in compliance with national guidelines under USDA APHIS import permits PCIP-17-00383 and PCIP-20-00092. Samples were dried down, homogenized and weighed for stable isotope analysis at the Stable Isotope Laboratory of the University of California, Santa Cruz. Nitrogen (%N) and carbon content (%C) were measured alongside δ^13^C and δ^15^N via a Dumas combustion using a 1108 elemental analyzer (Carlo Erba, Chaussée du Vexin, France) coupled to a Delta Plus XP isotope ratio mass-spectrometer (Thermo-Finnigan, Bremen, Germany). For the analysis of bulk leaf material δ^18^O we used a Thermo-Chemical Elemental Analyzer (Thermo-Finnigan, Bremen, Germany) also coupled to a Delta Plus XP isotope ratio mass-spectrometer. The resulting isotope data is reported here in ‰ (or mUr). Analytical precision was better than 0.2‰ for δ^13^C and δ^15^N, and better than 0.3‰ for δ^18^O.

We measured LMA in initially dried intact leaves, which were later rehydrated at the University of California, Santa Cruz. Dry leaves were weighed prior to rehydration and leaf area was measured using a LI-3100 Area Meter (cm^2^; LI-COR inc. Lincoln, Nebraska USA).

Using four linear mixed effects models we statistically analyzed the effects of height in interaction with PAR, tree species, %N and %C and the random effect of plant individual on the response variables δ^13^C, δ^18^O, δ^15^N and LMA (please see the Supplementary Materials for detailed material and method descriptions).

## Supplementary Information

Supplementary Information 1.

Supplementary Information 2.

Supplementary Information 3.

## Data Availability

All data generated or analyzed during this study are included in this published article as Supplementary Information file.
