# Supplementary material for: Stratigraphy of stable isotope ratios and leaf structure within an African rainforest canopy with implications for primate isotope ecology
Source: Sci Rep. 2021 Jul 9;11:14222. doi: 10.1038/s41598-021-93589-8 (PMC8270916; doi:10.1038/s41598-021-93589-8)
Supplement: Supplementary file 1 — Supplementary Information 1. [file 41598_2021_93589_MOESM1_ESM.docx]

**Supplementary material**

**Stratigraphy of stable isotope ratios and leaf structure within an African rainforest canopy with implications for primate isotope ecology**

Lowry, B.E.^1,2^, Wittig, R.M.^3,4^, Pittermann J.^2^, Oelze, V.M.*^1^

^1^ Department of Anthropology, University of California Santa Cruz, 1156 High Street, Santa Cruz, CA 95060, USA

^2^ Department of Ecology and Evolutionary Biology, University of California Santa Cruz, 1156 High Street, Santa Cruz, CA 95060, USA

^3^ Max Planck Institute for Evolutionary Anthropology, Deutscher Platz 6, 04103 Leipzig, Germany

^4^ Taï Chimpanzee Project, *Centre Suisse de Recherches Scientifiques*, B.P. 1303, Abidjan 01, Côte d’Ivoire

* Corresponding author: Vicky M. Oelze, voelze@ucsc.edu

**Materials**

Leaves were collected within the primary forest of Taï National Park in Western Côte d'Ivoire. The park is a continuous block of evergreen lowland rainforest along the coast of West Africa, between 6° 20’ N to 5° 10’ N and 4° 20’ W to 6° 50’ W. The park has a mean annual temperature of 24° C, with 85% relative humidity and mean annual rainfall of 1,830mm. Rainfall follows a four-season climate with two dry seasons (July to August) and (November to February) in-between two rainy seasons^1,2^.

For sampling we selected several individual leaves from the 12 predominant plant species from which leaves are particularly relevant for the diet of both chimpanzees (*Pan troglodytes versus*) and the different arboreal primates of Taï National Park. We used long-term feeding data (1997-2015) for the North and East group of the Taï Chimpanzee Project^3^ to determine the most common leaf species which chimpanzees frequently consume throughout the year (unpublished data, Boesch, Wittig et al.). We referred to the literature to also include leaf species regularly consumed by the Taï colobines (*Procolobus badius*, *Procolubus polycomos*) and other monkeys^1^ personal communications McGraw). Among these resulting 25 plant species we selected those species for which several trees were already identified and regularly monitored during phenology surveys. The 12 species which we sampled can be classified as were as emergent trees (n=4), midcanopy trees (n=3), lianas (n=2) and understory plants (n=3) (see Table 1).

**Methods**

*Sample and data collection*

We sampled 321 leaves from 58 individual plants of 12 different species at various locations throughout the Taï Chimpanzee Project research area^4,5^ in the relatively dry months between July and August of 2017 (see Table S1). VO used rope-based tree climbing strategies with the invaluable assistance, guidance and labor of arborist James Luce to safely and non-destructively reach the highest parts of the forest canopy, including the crowns of emergent trees^6,7^. While tree climbing was used for sample collection from trees and vines, sampling of understory plants was conducted from the ground. Although many primates prefer young over mature leaves, we decided to only sample mature leaves to standardize the developmental stages of leaves across individual plants sampled. Starting from the very tip of the crown for each tree, we first sampled the highest leaves at the very top of the tree crown and then gradually descended while systematically sampling leaves at approximately 1m (~3ft) intervals until reaching the base of the respective tree’s crown. The exact height of each sample was recorded using a measurement tape lowered to the ground using a weight bag. As a result, we collected on average approximately 6 leaf samples per individual tree (mean = 6.4 ± 1.5 1σ). Understory plants were sampled from the ground following 1m intervals as well, from the plant’s highest point to the forest floor.

Each leaf sample was associated with an instantaneous measure of photosynthetically active radiation (PAR; μmol m^-2^ s^-1^) using a quantum light sensor (LI-250A light meter and LI-190R-BNC-2 Quantum sensor, Li-Cor Biosciences, Lincoln, Nebraska). The unit was held over each leaf at three positions for 15 seconds each, and these data were used to compute the average PAR level, allowing us to account for slight changes in light conditions due to movements in the canopy. Leaves were clipped following each PAR measurement. Other factors obviously affecting PAR conditions during sampling were also recorded. Before we started sampling a plant we visually estimated percent cloud cover from the tree canopy or from the ground, as applicable, and recorded the time of day (see Table S1). Fluctuation in cloud cover, time of day, or season can greatly impact measurable PAR incident on a leaf. We are aware that due to changes in cloud cover and time of day, the instantaneous light readings we took may not be representative of the full spectrum of light intensity a leaf might receive during a single day or over the course of a year. However, we are confident that our instantaneous light measurements are representative because PAR was collected for each leaf under similar overcast conditions and around midday (Table S1).

Every sample, consisting of multiple leaves, was placed into a paper envelope and dried onsite by first using an oven at low heat and subsequent storage on silica gel. Samples were then shipped to the Primate Ecology and Molecular Anthropology (PEMA) lab at the University of California, Santa Cruz for analysis under the USDA controlled import permit PCIP-17-00383 (2020 extension PCIP-20-00092).

*Stable isotope analysis*

We homogenized approximately 1g of fully dried leaves by cutting small amounts of leaf material from all leaves represented in one sample and reducing the material to a fine powder in a ball mill (Mixer Mill MM40, Retsch Technology, Haan, Germany). Past studies of isotopic values of leaf water have found spatial patterns of increasing enrichment in certain areas within a leaf^8,9^. To control for spatial δ^18^O heterogeneity within leaves, we obtained our samples by cutting from numerous positions on each leaf.

For simultaneous δ^13^C and δ^15^N analysis, 2700 to 3000 µg of homogenized leaf matter was weighed into 5x9 mm tin capsules using a microbalance (Model XS3DU, Mettler Toledo, Columbus, Ohio). All isotopic composition was measured by Dumas combustion using a 1108 elemental analyzer (Carlo Erba, Chaussée du Vexin, France) coupled to a Delta Plus XP isotope ratio mass-spectrometer (Thermo-Finnigan, Bremen, Germany) at University of California, Santa Cruz Stable Isotope Laboratory. Analytical precision for oak, pugel, and acetanilide standards was better than 0.2‰ for δ^13^C and δ^15^N. Elemental composition was determined based on IRMS traces and provided data on carbon (%C) and nitrogen (%N) contents of each sample

For analysis of bulk leaf δ^18^O (rather than leaf water), 40 to 60 µg of homogenized leaf matter was deposited in silver capsules for measurement on a continuous flow TC/EA IRMS analysis. The Thermo-Chemical Elemental Analyzer (TCEA) uses pyrolysis to reduce hydrogen at 1400ºC and separates H_2_ and CO by chromatography in a He gas stream. No catalyst material was required. Elemental composition of samples was calibrated by three benzoic acid standards, Hekatek, IAEA601 and IAEA602 reported relative to VSMOW^10,11^. Analytical precision for Hekatek and IAEA601 standards was better than 0.3‰, IAEA602 standard is used for scale normalization and so precision is not used as a reference for sample quality. Exchangeable oxygen was not quantified in this study, but samples were stored on silica in the field and in a desiccator in the lab, particularly when processed and weighed to avoid exchange with air oxygen.

*Leaf Mass Per Area*

  Usually leaf mass per area is measured using fresh leaves but this was not considered feasible due to practical constraints. Instead, the dried leaves were rehydrated at the University of California, Santa Cruz, and the leaf area was measured using a LI-3100 Area Meter (cm^2^; LI-COR inc. Lincoln, Nebraska, USA). This method of rehydrating already desiccated leaves in pure water has been seen to significantly reverse leaf shrinkage due to drying^12^.

We measured within site variation in LMA for the majority of leaf samples collected from Taï forest (n = 214). We were not able to effectively rehydrate leaves for all samples either because there were not enough intact leaves to accurately measure LMA or because the structure of the leaf was too fragile to withstand rehydrating. Some leaves such as those of the woody legume *Parka bicolor* were too fragmented after the drying process that they could not be used for LMA measurements. For the remaining taxa, up to three leaves were selected per sample for intactness and representative size. Dry mass was recorded in mg for each leaf using an analytical balance (Entrisx-1S, Sartorius) then all leaves were subjected to the same rehydration procedure. Leaves were rehydrated in deionized water for 48 hours, then after rehydration, leaf area in cm^2^ was measured using the average of three consecutive measurements in a leaf area meter. Based on the leaf weights and area measurements we calculated the average LMA for each sample of leaves in g/m^2^.

*Statistical data analysis*

We tested the effect of sampling height, light exposure (PAR), nitrogen and carbon content of leaves as well as tree species on the stable isotope ratios and LMA measurements of leaves across the forest canopy. We excluded a single statistical outlier (Lan.2.1) for which a low C:N ratio and extreme δ^13^C value indicated sample contamination or measurement error.

We ran four linear mixed effects models with Gaussian error structure in R (version 3.6.3)^13^ using the lmer function (lme4 package^14^). Models were fitted by maximum likelihood tests and with significance levels set to 0.05. We ran a model each on the normally distributed response variables δ^13^C, δ^18^O and δ^15^N in the full dataset of 321 samples. The fourth model included the response LMA in a subset of 214 samples. To gain normalized distributions in the skewed predictor variables height and PAR we log-transformed height and implemented a log10 transformation on the variable PAR. The main predictor variables included the interaction between height (m) and PAR (μmol), %N, %C, tree species as well as the random effect (intercept and slopes) of individual ID, accounting for multiple measurements within the same individual plant^15^. In each model we tested for the significance of the interaction between the height and PAR covariates using an ANOVA. The interaction was found to be significant in all models except for the δ^15^N model, for which we removed the interaction in the resulting full model. We conducted model diagnostics by visually inspecting qq-plots and histograms of the residuals plotted against fitted values, both of which suggested normally distributed and homogeneous residuals in all four models (Table S2). We tested variance inflation factors from the results of standard linear models excluding interactions and random slopes and values consistently lower than 2.5 indicated no evidence for collinearity. We then evaluated model stability by excluding the levels of random effects one at a time and by comparing their results with the original models. Finally, all four model results were obtained by testing the full models compared to null models excluding the main effects using chi-square tests of independence.

**References**

1. McGraw, W. S. Comparative locomotion and habitat use of six monkeys in the Tai Forest, Ivory Coast. *Am J Primatol* **105**, 493–510 (1998).

2. Boesch, C., Boesch, P. C. & Boesch-Achermann, H. *The Chimpanzees of the Taï Forest: Behavioural Ecology and Evolution*. (Oxford University Press, 2000).

3. Gone Bi, Z. B. & Wittig, R. M. *Long-term diet of the chimpanzees (Pan troglodytes verus) in Taï National Park: inter-annual variations in consumption of some key food sources*. *The chimpanzees of the Taï forest: 40 years of research* 242–260 (Cambridge University Press, 2019).

4. Wittig, R. M. Taï Chimpanzees. in *Encyclopedia of Animal Cognition and Behavior* (eds. Vonk, J. & Shackelford, T.) 1–7 (Springer International Publishing, 2017). doi:10.1007/978-3-319-47829-6_1564-1.

5. *The Chimpanzees of the Taï Forest: 40 Years of Research*. (Cambridge University Press, 2019). doi:10.1017/9781108674218.

6. Anderson, D. L., Koomjian, W., French, B., Altenhoff, S. R. & Luce, J. Review of rope-based access methods for the forest canopy: safe and unsafe practices in published information sources and a summary of current methods. *Methods in Ecology and Evolution* **6**, 865–872 (2015).

7. Anderson, D. L. *et al.* Can increased training and awareness take forest research to new heights? *Trees, Forests and People* **1**, 100005 (2020).

8. Helliker, B. R. & Ehleringer, J. R. Establishing a grassland signature in veins: 18O in the leaf water of C3 and C4 grasses. *Proceedings of the National Academy of Sciences* **97**, 7894–7898 (2000).

9. Cernusak, L. A. *et al.* Stable isotopes in leaf water of terrestrial plants. *Plant, Cell & Environment* **39**, 1087–1102 (2016).

10. Brand, W. A. *et al.* Comprehensive inter-laboratory calibration of reference materials for delta O-18 versus VSMOW using various on-line high-temperature conversion techniques. *Rapid Communications in Mass Spectrometry*, **23**, 999–1019 (2009).

11. Coplen, T. B. *et al.* New guidelines for delta C-13 measurements. *Analytical Chemistry*. **78**, 2439–2441 (2006).

12. Scoffoni, C., Vuong, C., Diep, S., Cochard, H. & Sack, L. Leaf Shrinkage with Dehydration: Coordination with Hydraulic Vulnerability and Drought Tolerance. *Plant Physiol.* **164**, 1772–1788 (2014).

13. R Core Development Team. R: A language and environment for statistical

computing. R Foundation for Statistical Computing, Vienna, Austria. <http://www.R->

project.org/. (2013).

14. Bates, D., Mächler, M., Bolker, B. & Walker, S. Fitting Linear Mixed-Effects Models Using lme4. *Journal of Statistical Software* **67**, 1–48 (2015).

15. Hurlbert, S. H. Pseudoreplication and the Design of Ecological Field Experiments. *Ecological Monographs* **54**, 187–211 (1984).
